# Motivational Interviewing and Medication Review in Coronary Heart Disease (MIMeRiC): Protocol for a Randomized Controlled Trial Investigating Effects on Clinical Outcomes, Adherence, and Quality of Life

**DOI:** 10.2196/resprot.8659

**Published:** 2018-02-20

**Authors:** Malin Johansson Östbring, Tommy Eriksson, Göran Petersson, Lina Hellström

**Affiliations:** ^1^ Pharmaceutical Department Kalmar County Council Kalmar Sweden; ^2^ eHealth Institute Department of Medicine and Optometry Linnaeus University Kalmar Sweden; ^3^ Department of Biomedical Science Malmö University Malmö Sweden; ^4^ Department of Clinical and Molecular Medicine Norweigan University of Sciences and Technology Trondheim Norway

**Keywords:** medication adherence, medication therapy management, pharmacist, coronary artery disease, randomized controlled trial

## Abstract

**Background:**

Preventive treatment goals for blood pressure and cholesterol levels continue to be unmet for many coronary patients. The effect of drug treatment depends on both its appropriateness and the patients’ adherence to the treatment regimen. There is a need for adherence interventions that have a measurable effect on clinical outcomes.

**Objective:**

This study aims to evaluate the effects on treatment goals of an intervention designed to improve patient adherence and treatment quality in secondary prevention of coronary heart disease. A protocol for the prespecified process evaluation of the trial is published separately.

**Methods:**

The Motivational Interviewing and Medication Review in Coronary heart disease (MIMeRiC) trial is a prospective, randomized, outcomes-blinded trial designed to compare individualized follow-up by a clinical pharmacist using motivational interviewing (MI) and medication review with standard follow-up. Patients were randomized to 2 groups after stratification according to their beliefs about medicines. After standard follow-up at the cardiology clinic, patients in the intervention group are seen individually by a clinical pharmacist 2 to 5 times as required over 7 months, at the clinic. The pharmacist reviews each patient’s medication and uses MI to manage any problems with prescribing and adherence. The primary study outcome is the proportion of patients who have reached the treatment goal for low-density lipoprotein cholesterol by 12 months after discharge. Secondary outcomes are the effects on patient adherence, systolic blood pressure, disease-specific quality of life, and health care use.

**Results:**

The protocol for this study was approved by the Regional Ethics Committee, Linköping, in 2013. Enrollment started in October 2013 and ended in December 2016 when 417 patients had been included. Follow-up data collection will conclude in March 2018. Publication of the primary and secondary outcome results from the MIMeRiC trial is anticipated in 2019.

**Conclusions:**

The MIMeRiC trial will assess the effectiveness of an intervention involving medication reviews and individualized support. The results will inform the continued development of support for this large group of patients who use preventive medicines for lifelong treatment. The design of this adherence intervention is based on a theoretical framework and is the first trial of an intervention that uses beliefs about medicines to individualize the intervention protocol.

**Trial Registration:**

ClinicalTrials.gov NCT02102503; https://clinicaltrials.gov/ct2/show/NCT02102503 (Archived by WebCite at http://www.webcitation.org/6x7iUDohy)

## Introduction

Coronary heart disease (CHD) is the leading cause of death worldwide, and an aging population means that the number of people affected by the disease is increasing [1]. The acute treatment of CHD has been revolutionized in the last two decades, and mortality and morbidity have been more than halved [2,3]. This means that more patients are now treated with secondary prevention measures to minimize the risk of new CHD events. Pharmacological treatment for secondary prevention of CHD reduces morbidity and mortality through a direct thromboprophylactic effect and through effects on hypertension, hyperlipidemia, and high blood glucose, with resultant reductions in the progression of atherosclerotic plaque and stabilization of plaques. The effect of the drug treatment depends on both its appropriateness to the individual and the patient’s adherence to the dosage regimen. Suboptimal prescribing and poor adherence increase morbidity and mortality [4]. Despite established guidelines and widespread access to effective and inexpensive medicines, preventive treatment goals for blood pressure and cholesterol continue to be unmet for many coronary patients [5-7]. The reasons for this include suboptimal prescribing and the 20% to 30% of patients who stop taking their preventive medicines, that is, whose adherence worsens, at some point after the initiation of treatment [4,8]. In a report on the burden of nonadherence, the World Health Organization concluded that “Increasing the effectiveness of adherence interventions may have a far greater impact on the health of the population than any improvement in specific medical treatments” [9].

The reasons for nonadherence are multiple and individual, and therefore, any attempted intervention must have a broad approach to inventorying problems and must allow for individualized problem solving to be effective in a wide group of patients [9]. Interventions that are effective for both adherence and clinical outcomes are usually complex in nature, according to a Cochrane review [10]; however, overall, there is little evidence that adherence interventions can enhance clinical outcomes [11]. This is in part because the studies often lack the power to detect differences in clinical outcomes and sometimes also in the adherence outcome [11]. Another large review and meta-analysis of 771 adherence interventions, which did not include clinical outcomes, suggests that interventions may have a small effect on adherence and that this effect is higher for interventions delivered face-to-face, by pharmacists, and with a behavioral rather than a cognitive approach [12]. A Cochrane review of adherence interventions for lipid-lowering drugs also suggests that team-based intensification of patient care can improve cholesterol management through better adherence in both short and long term [13].

A recent review of interventions for patients with CHD suggests that simple adherence interventions might be as effective as complex ones, but this review only studied effects on adherence, and in half of the included studies, patients were followed up only for 6 months or less [14]. Adherence to the right medicines must increase for the intervention to be effective, and pharmacist interventions (including patient education, feedback to the physician, and medicine management) can improve risk factor management in patients with cardiovascular disease [15-18]. Motivational interviewing (MI) has been used with some effect in medication adherence interventions [19-21] and also specifically when administered by nurses in cardiac care [22].

The theoretical framework for the intervention evaluated in this study is described in detail in a separate manuscript, which also describes the development from pilot study and the study protocol for evaluation of the intervention process [23].

The primary objective of this trial was to evaluate the effects of MI and a medication review, as part of a secondary prevention program in patients with CHD, on achieving goal levels of low-density lipoprotein cholesterol (LDL-C) by 12 months after discharge, compared with standard care.

The secondary objectives were to evaluate the effects of the intervention on systolic blood pressure, adherence to secondary prevention drugs, health-related quality of life (general and disease-related), and secondary care use. A health economic assessment will also be conducted, but this is not described in detail in this study protocol.

## Methods

### Trial Design

Motivational Interviewing and Medication Review in Coronary heart disease (MIMeRiC) is a randomized, controlled, outcomes-blinded, superiority trial with two parallel groups. Patients have been randomized to standard care (control) or standard care plus a follow-up program that includes medication review and MI (intervention). Ethical approval has been obtained from the Regional Ethics Committee, Linköping, Sweden (Dnr-2013/236-31). The trial is registered in clinicaltrials.gov (NCT02102503).

### Study Setting and Population

Patients with CHD (International Classification of Diseases-10 I20-I21) were recruited from the cardiology unit at the County Hospital in Kalmar, Sweden. This is a 400-bed teaching hospital in rural Sweden; the cardiology unit has 30 beds and performs around 1300 angiographies and 600 percutaneous coronary interventions a year, but no open-heart surgery. All patients with coronary artery disease, regardless of how acute it was, were chosen because they all undergo the same standard follow-up at the outpatient clinic. See Textbox 1 for inclusion and exclusion criteria.

**List of inclusion and exclusion criteria.**

Inclusion criteria:

Patients must:

- speak Swedish
- have an angiography during their hospital stay
- be scheduled for follow-up at the out-patient clinic in Kalmar
- have verified coronary artery disease (International Classification of Diseases-10 I20-I21)

Exclusion criteria:

Patients are excluded if any of the following conditions apply:

- cognitive impairment or any other condition making interviews or phone calls difficult
- nonparticipation in the standard follow-up at the outpatient clinic
- prior participation in this study

### Recruitment

The recruitment of patients was changed during the study because of practical problems with screening. This change was judged not to affect the generalizability of the result, and it was verified by the Regional Ethics Committee.

#### October 2013-May 2014, November 2014-September 2015

Patients admitted to the coronary angiography unit were screened for eligibility, and eligible patients were given written and verbal information about the trial by a nurse or a study pharmacist and were invited to take part. Patients who agreed to participate were contacted within 2 weeks by a pharmacist who explained the implications of the research and asked for informed consent (documented by the pharmacist during the phone call).

#### October 2015-December 2016

Patients scheduled for a follow-up visit to a cardiology nurse 2 weeks after discharge were screened for eligibility. Eligible patients were given written information to read in the waiting room, and verbal information was given by the nurse. The nurse explained the implications of the research and asked for written informed consent.

### Randomization

**Figure 1:**
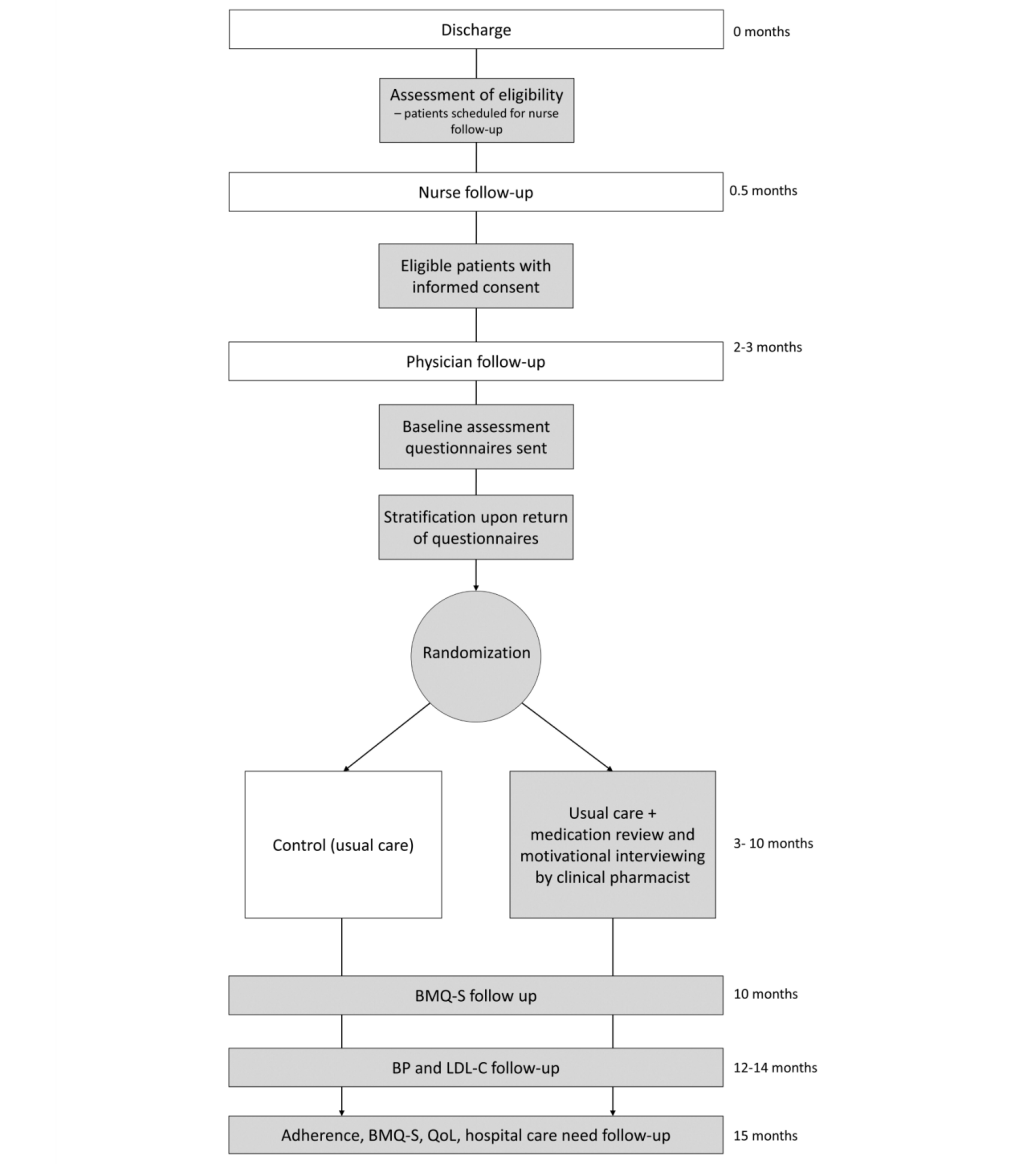
Study flow chart. Each box represents a separate event. White boxes are standard care events, light gray boxes are study events; BMQ-S: Beliefs about Medicines Questionnaire-Specific; BP: blood pressure; LDL-C: low density lipoprotein cholesterol; QoL: quality of life.

The patients were randomized in blocks of 10, stratified according to their attitudes toward their heart medicines, as measured by the Beliefs about Medicines Questionnaire-Specific (BMQ-S) [24,25] after their standard care follow-up with the physician (see Figure 1). Patients may be accepting (A), ambivalent (B), or neutral or skeptical (C), which can affect their likelihood of being adherent [26,27]. An accepting patient has a strong belief in the necessity for drugs and minimal concerns about the drug, an ambivalent patient has a strong belief in the necessity for drugs and is highly concerned about the drug, and a skeptical patient does not believe in the necessity for drugs and is highly concerned about the drug. We chose to stratify the patients according to their attitude toward medication because a patient’s beliefs about medication are partly affected by their previous medical history and type of CHD, and we believe that the patient’s attitude toward medication has a greater effect on adherence and the need for intervention than these underlying factors. Data on patient beliefs and other baseline measures were collected directly after the physician visit. The randomization sequence for each stratum was computer-generated by a statistician who is not involved in data collection. An intervention to control allocation ratio of 1:1.14 was chosen to account for an expected greater loss to follow-up in the control arm. For each patient, a folded sheet of paper with the group allocation and unique study identification number written on it was kept in a sealed opaque envelope marked with a serial number; it was impossible to read the information without opening the envelope. A study administrator collected baseline questionnaires and defined the stratum of each patient before assigning the patient to the intervention or control groups according to the serial number.

### Intervention Group Protocol

The intervention is a follow-up program run by a clinical pharmacist, which is carried out in addition to the standard care. The clinical pharmacist carries out MI and reviews the patient’s medication. The mainstay of the intervention consists of two appointments at the cardiac outpatient clinic, but this is adjusted according to the patient’s needs. For a full list of study events, see Table 1.

#### First Visit, 3 Months After Discharge

Intervention participants are scheduled for a 60-min appointment with the clinical pharmacist, following their standard follow-up appointments at the clinic, around 3 months after discharge. The pharmacist prepares an advanced medication review [28] based on documentation in the electronic health record (EHR) that is shared between the hospital and primary care facility, applying national and European guidelines to assess the quality of prescribing [29,30]. The baseline data on the patient’s beliefs about medicines are also recorded.

The clinical pharmacist uses MI when seeing the patient. An agenda is set to focus the interview on how the medication works for the patient, what it means in terms of side effects, the patient’s worries, their understanding of the purpose of the medicines, and their thoughts about risks and benefits. The goal is that the patient should feel safe and secure with their medication, and that any problems affecting adherence or quality of life will be found and solved together. If the medication review indicates a need for intensifying the treatment, this is first discussed with the patient to assess their readiness for change. At the end of the consultation, the pharmacist prepares a written summary of the discussed issues and the agreed next steps. The summary is given to the patient together with the next scheduled appointment time.

Any drug-related problems that cannot be solved by the pharmacist and patient together are discussed with the cardiologist after the visit either in person or via the EHR, and the pharmacist then contacts the patient by phone if prescription changes are made. The pharmacist documents the assessment and findings in the EHR.

**Table 1:** Schedule of events by treatment arm.

Study event (including standard care)		Control	Intervention
Discharge		Always	Always
Nurse visit (2 weeks after discharge)		Always	Always
Physical training in cardiac rehabilitation is offered		Always	Always
Referral to welfare officer		If needed	If needed
Physician visit (around 2 months after discharge)		Always	Always
Extended follow-up in cardiac clinic or primary-care facility		If needed	If needed
Referral to primary-care facility		Always	Always
**First pharmacist visit (around 3 months after discharge)**			
	Medication review and MI^a^		Always
	Written summary of discussion		Always
	Discussion with cardiologist if problems with cardiac drugs or treatment goals		If needed
	Referral to primary-care facility if problems with other drugs		If needed
	Follow-up phone call		Always
**Intensified intervention only**			
	Up to four extra contacts by phone or in person		If there are negative attitudes or drug-related problems^b^
**Follow-up pharmacist visit (10 months after discharge)**			
	Medication review and MI		Always
	Written summary of discussion		If needed
	Follow-up phone call		If needed
	Referral to primary-care facility if problem with any drug or treatment goal		If needed

^a^MI: motivational interviewing.

^b^Intensified intervention is not a randomized treatment arm.

The pharmacist makes a follow-up phone call 2 weeks after the visit to enquire about the agreed changes, to see if there are new questions, and to strengthen the message from the interview.

#### Follow-Up Visit, 10 Months After Discharge

The pharmacist reviews the EHR for any changes in health and prescribing, and monitors the lipid profile (the patient receives a referral for a laboratory test along with the scheduled appointment), before seeing the patient. The patient’s beliefs about medicines are reassessed, and MI is used to elicit the patient’s thoughts and problems. The consultation, which lasts approximately 20-30 min, aims to support the patient for their coming lifelong (supposedly) medicine use and to guide them to obtain follow-up at a primary care facility if they have no established primary care contact. Any problems found at this stage are communicated to the primary care physician, either through referral or with a personal message in the EHR. A written summary is provided and a follow-up phone call is made only if new problems are encountered.

#### Adjusting the Intervention According to the Patient’s Need

The intervention protocol is adjusted according to the patient’s beliefs about medicines or need for support. If the patient is assessed as accepting at baseline, the pharmacist can shorten the initial consultation to 30-40 min if appropriate. If the patient has negative beliefs, that is, ambivalent, skeptical or neutral, the pharmacist arranges a more thorough interview and offers the patient more visits or continued contact by phone. This more intensive intervention protocol offers the patient up to four extra contacts, either in person or by phone, as an extension of the first visit. During the first visit, the pharmacist and the patient decide together whether the patient’s worries or drug-related problems require more contacts.

#### Intervention Pharmacists

The intervention is performed by two clinical pharmacists (LH and MJÖ) with training in both medication review and MI. One of the pharmacists has formal specialist training in clinical pharmacy, focusing on cardiovascular medicine (60-credit Master’s program in clinical pharmacy at Uppsala University, Sweden) and has completed a 15-credit course in MI from Linnaeus University, Sweden. The other has completed a 12-credit course in clinical pharmacy and pharmacotherapy from Lund University, Sweden, 2 days of internal training in MI, and a 3-day course run by a member of Motivational Interviewing Network of Trainers. Both pharmacists have carried out 5 consultations coded by Motivational Interviewing Treatment Integrity 3.1 with feedback and, in at least one of these, have been evaluated as “beginning proficiency” (≥3.5) in the global rating of MI-spirit.

### Standard Care

Participants in the control group receive standard care only. Standard care at the cardiology unit of the County Hospital in Kalmar comprises a 60-min appointment with a cardiac specialist nurse 2 weeks after discharge and a 60-min appointment with an assistant physician or cardiologist about 2 months after discharge. Unless the patient requires specialist follow-up or more treatment at the cardiac clinic, referral is made to the primary care facility for continuing follow-up. All patients are also offered cardiac rehabilitation such as physical training in a group at the hospital or at a primary care facility closer to home. See Table 1 for details.

### Study Parameters and Data Collection

Baseline assessment data, including demographics, level of education, civil status, CHD presentation type, previous CHD history, comorbidities, smoking status, type of cardiovascular intervention, and prescribed medicines, were collected from the EHR by a member of the research staff before randomization. Further baseline data were obtained from questionnaires sent to the participants by mail after their physician visit at the cardiology clinic with instructions to return them within 10 days; these questionnaires covered medication adherence, beliefs about medicines, and health-related quality of life. Baseline data on lipid status and blood pressure were collected from the EHR. See Table 2 for a full list of the collected data and Figure 1 for an outline of study assessments.

To promote participant retention, control group patients receive a postal card stating the appreciation of the research team for their return of questionnaires at baseline and 10 months. Intervention group patients do not receive a card as they are instead summoned for a visit.

Lipid status and blood pressure are assessed 12-14 months after discharge. Patients who have had a myocardial infarction are followed-up at 12-14 months by the national quality register SEPHIA (Secondary prevention after Heart Intensive care Admission), and assessments of lipids and blood pressure are therefore recorded in the EHR. We use these data so that participants do not need an extra assessment because of the study. For noninfarction patients, the research team arranges for the assessment of lipids and blood pressure. All patients contacted by either SEPHIA or the research team receive a referral for a laboratory test and blood pressure measurement, which they can choose to do at the drop-in clinic at the hospital or at their primary care facility.

At 15 months postdischarge, participants complete all questionnaires for the outcomes assessment: Morisky 8-item adherence scale (MMAS-8), BMQ-S, EuroQoL 5 Dimensions 5 Levels, and HeartQoL questionnaire. A time of 15 months was chosen to relate the answers on the MMAS-8 to the pharmacy refill date 12-16 months after discharge. Data are collected from the Swedish Drug Prescription Register (adherence), the Health Care Register of Kalmar County (hospital admissions), and, for deceased participants, the registry of Causes of Death administered by the National Board of Health and Welfare.

### Control for Bias

Because randomization took place after the standard care process, the doctors and nurses involved in these standard visits did not know whether their patients would be in the control or the intervention group. This introduced a control for bias during the standard care period. However, this control is lost for doctors with whom the pharmacist discusses treatment during subsequent periods of the study, as it will be obvious that they are discussing intervention patients, and for doctors and nurses involved in the care of those intervention patients who have further contact with the clinic after the standard follow-up. Pharmacists are not involved in any care of patients at the cardiology clinic outside of this study.

All the outcomes data (returned questionnaires, prescription fill data, and health care use) for each patient are collected in an individual, coded, clinical research form (CRF). Data from registers and the EHR are collected by a blinded research assistant who is not involved with the care of the study patients. Researchers will enter the data from the coded CRFs into the database.

To assess selection bias, all participants will be compared with eligible patients who declined to participate, in terms of age, sex, type of CHD, new or recurrent CHD, and marital status.

### Statistical Methods

The primary analysis will take place 16 months after inclusion of the last patient, according to the intention-to-treat principle. The primary outcome will be analyzed using logistic regression models. Secondary outcomes will be analyzed with appropriate statistical methods based on the type of data. Primary and secondary regression analyses will be adjusted for baseline variables. Per protocol analyses will also be performed. All tests will be two-sided and a *P* value of <.05 will be considered significant.

### Sample Size

#### Initial Assumptions and Calculations

In quality registry data from 2012, the proportion of patients achieving the LDL-C treatment goal in Kalmar was less than 0.3 [33]. To detect a shift in proportion from 0.3 to 0.5 in goal achievement for LDL-C, our initial sample size calculation resulted in a group size of 93 patients, for 80% power at a significance level of *P*=.05 (two-sided).

**Table 2:** Study assessment schedule indicating when data is collected.

Collection of data		Discharge	Nurse visit at 2 weeks	Physician visit at 2 months	Baseline questionnaire^a^	10 months	12 months	15 months
Patient eligibility			✓					
Patient informed consent			✓					
**Retrospectively after consent (EHR**^b^**)**								
	Patient medical history	✓						
	Demographics	✓						
	Medications	✓						✓
	Lipid panel	✓		✓			✓	
Systolic blood pressure (EHR)				✓			✓	
BMQ-S^c^					✓	✓		✓
**QoL**^d^**, health-related**								
	Heart-QoL				✓			✓
	EQ-5D-5L^e^				✓			✓
**Medication adherence**								
	Self-reported				✓			✓
	Pharmacy refill							✓
Hospital admissions								✓

^a^Sent after the physician visit.

^b^EHR: electronic health record.

^c^BMQ-S: Beliefs about Medicines Questionnaire-Specific.

^d^QoL: quality of life.

^e^EQ-5D-5L: EuroQoL questionnaire [31,32].

Another registry, the national “Öppna Jämförelser” (Open Comparisons), measures the proportion of patients who have had a myocardial infarction and who fill a prescription for a statin 12-16 months later. The report from 2012 stated that 80% of myocardial infarction patients from Kalmar County Hospital filled a statin prescription [34]. To detect a difference of 10% in the proportion of patients with refill adherence, with 80% power at a significance level of *P*=.05 (two-sided), 195 patients would be required in each group.

We assumed an attrition rate of 40% in the intervention group and 60% in the control group, because the protocol for the latter can be regarded as an extended questionnaire study. Because patients were enrolled about 2 months before they were asked to fill in the first set of questionnaires, we assumed a high attrition rate at this stage, and because they are volunteers, we wanted withdrawal from the study at this stage to be a simple process. Patients who did not answer these first questionnaires will not be included in the outcome analyses.

On the basis of our primary outcome (LDL-C goal achievement) and expected attrition rate, a sample size of 130+140 patients (intervention plus control) would be required. However, this would not have the power to detect a meaningful difference in adherence (one of the secondary outcomes). As one of the problems encountered in prior intervention studies has been the lack of power to detect differences in both adherence and clinical outcomes, we based our sample size calculation on the number required to show a difference in adherence, that is, 195 patients at follow-up.

We therefore aimed to include 273+312 (=585) patients in the intervention and control groups, with an allocation ratio of 1:1.14.

#### Amended Sample Size Calculation in 2016

During the study, we learned two things that greatly impacted our sample size: (1) the goal achievement in standard care improved significantly and (2) our assumed attrition rate was too high. As described earlier, problems with recruitment also delayed the study, and this was another incentive to look at the required sample size.

The goal achievement for LDL-C in 2012 did not reflect the circumstances during our follow up in 2014-2017 because treatment possibilities changed the likelihood of reaching the target (the atorvastatin patent expired in 2013 and local guidelines successively changed, based on this). In 2015, the proportion of patients reaching the target was 0.5 nationally and 0.45 at Kalmar Hospital [35]. This reduced the power of the study to reject the null hypothesis unless the sample size was increased. On the other hand, our estimated attrition rate of 40-60% was shown to be too high, as only 16.9% (71/418) patients failed to return their baseline questionnaires, and with exclusions for other reasons (after obtaining patient consent), our attrition rate was 23% up to baseline assessment. We calculate the attrition based on this because all patients who filled in the baseline questionnaires, who do not later contact us to withdraw their consent, can be assessed for the primary outcome (LDL-C) as well as the pharmacy fill adherence and hospital admission outcomes, even if they drop off and fail to return follow-up questionnaires. As the assumptions behind the sample size calculation for the adherence measure are very uncertain and our funding would not permit recruitment after the end of 2016, we prioritized power for the primary outcome, and it was decided to end recruitment when at least 400 patients had consented or in December 2016 at the latest. The new calculation was based on the goal achievement of 0.45 at Kalmar Hospital and our expectation to reach 0.6 in the intervention group. This would mean 170 patients needed in each group for a power of 80% to reject the null hypothesis, or 134 needed for 70% power.

### Outcomes

#### Primary Outcome

The primary outcome parameter of the MIMeRiC trial is the proportion of patients who reach the treatment goal for LDL-C levels. The treatment goal, as assessed by SEPHIA, is an LDL-C of <1.8 mmol/L, or a reduction of 50% from baseline.

LDL-C was chosen as the primary outcome because it is an objective measure of a variable related to the risk of recurrent disease. The national quality registry data indicate that it is more difficult to reach treatment goals for LDL-C than for systolic blood pressure [33], and we also regarded the measurement of LDL-C at a single laboratory to be more reliable than blood pressure measurements at several different health care facilities.

The assessment of LDL-C is part of the follow-up process in SEPHIA, and the test is administered by the cardiology outpatient clinic for all patients with acute myocardial infarction. Patients with other forms of CHD will be followed by the research team for this assessment and asked to go to their primary care facility or the cardiology outpatient clinic for assessment, whichever is most convenient for them. LDL-C values are calculated from the serum concentrations of cholesterol and fasting triglycerides, using the Friedewald formula.

#### Secondary Outcomes

##### Patient Adherence

The proportion of patients who adhere to the treatment regimen will be assessed using self-reporting and refill data. The phases of adherence under study are implementation and persistence, as defined by the ABC-taxonomy [36]. Because self-reporting and refill data have their individual disadvantages, they will be combined [37]; thus, the patient is considered nonadherent if either they are nonpersistent according to refill data or they are nonadherent according to self-report. However, as self-reporting is only possible for one medication per questionnaire [38], we will use this combined measure only for cholesterol-lowering drugs, as they relate to our primary outcome.

Self-reported adherence to cholesterol-lowering drug regimens will be measured with the MMAS-8 [38-40]. Although this has been validated in hypertension, studies have validated the earlier version (MMAS-4) in statin treatment [41,42]. Self-reporting with different methods or instruments has been used in adherence trials using MI [20] and in many studies of adherence interventions in general [11]. For the combined adherence measure, patients will be regarded as nonadherent in the implementation phase if they score <6 points on the MMAS-8 [37,39]. However, to further investigate the relationship between different adherence measures and the outcome, we will tabulate the results from the MMAS-8, refill adherence, and LDL-C assessments and perform statistical analyses, using the 3 categories of high (8), medium (6 to 7.75), and low (<6) adherence in MMAS-8 [38].

Refill adherence will be assessed using the Swedish Prescribed Drug Register. Patients will be defined as nonpersistent if they have not purchased the drug at least once during the 12- to 16-month period after discharge. The 4-month period is based on the Swedish reimbursement system [43]. The proportion of patients who are persistent to dosage regimens for cholesterol-lowering drugs, aspirin, platelet aggregation inhibitors, beta-blocking agents, angiotensin-converting enzyme inhibitors, and angiotensin receptor blockers will be assessed using refill data compared with prescription data, as recorded in the EHR. For cholesterol-lowering drugs, a third adherence estimate will be used, the percentage of days covered adherence measure. A cutoff point of 80% has been set for the percentage of days covered measure [44]. This means that if a patient has collected medicines during the follow-up period, but has not collected enough to cover 80% of the doses prescribed in the EHR, the patient is considered either nonadherent during implementation or as having discontinued treatment.

##### Systolic Blood Pressure

We will also measure the proportion of patients with systolic blood pressure <140 mm Hg 12 months after discharge. As for LDL-C, this is part of the second follow-up in SEPHIA. Patients not included in the SEPHIA registry will be followed by the research team and asked to attend their primary care facility or the cardiology outpatient clinic for assessment, whichever is most convenient for them.

##### Quality of Life

Changes in quality of life will be measured with the HeartQoL [45] questionnaire. This questionnaire was developed for use in patients with ischemic heart disease; it measures both physical and emotional items. Mean changes between baseline and follow-up will be calculated for each group, as well as the proportion of patients with increased, maintained, or decreased quality of life. Our rationale for measuring this is that the items of HeartQoL would be affected partly if the treatment is better but possibly more by reducing side effects. It might be that increased treatment or adherence has a negative impact or that a more individual treatment leads to improved quality of life because of fewer side effects. However, there could also be negative consequences on the preventive effect for CHD. Although quality of life is challenging to evaluate, and multiple instruments can be used, there is reason to believe that this could be an important outcome in adherence interventions [46].

##### Secondary Care Use

Information about the patients’ unscheduled secondary care use will be collected from the health care register in the County of Kalmar. The number of emergency visits or hospitalizations due to cardiovascular disease and the time to first contact will be recorded for each group. Data will also be collected retrospectively for the 10-year period before the index date so that adjustments can be made as required.

## Results

**Figure 2:**
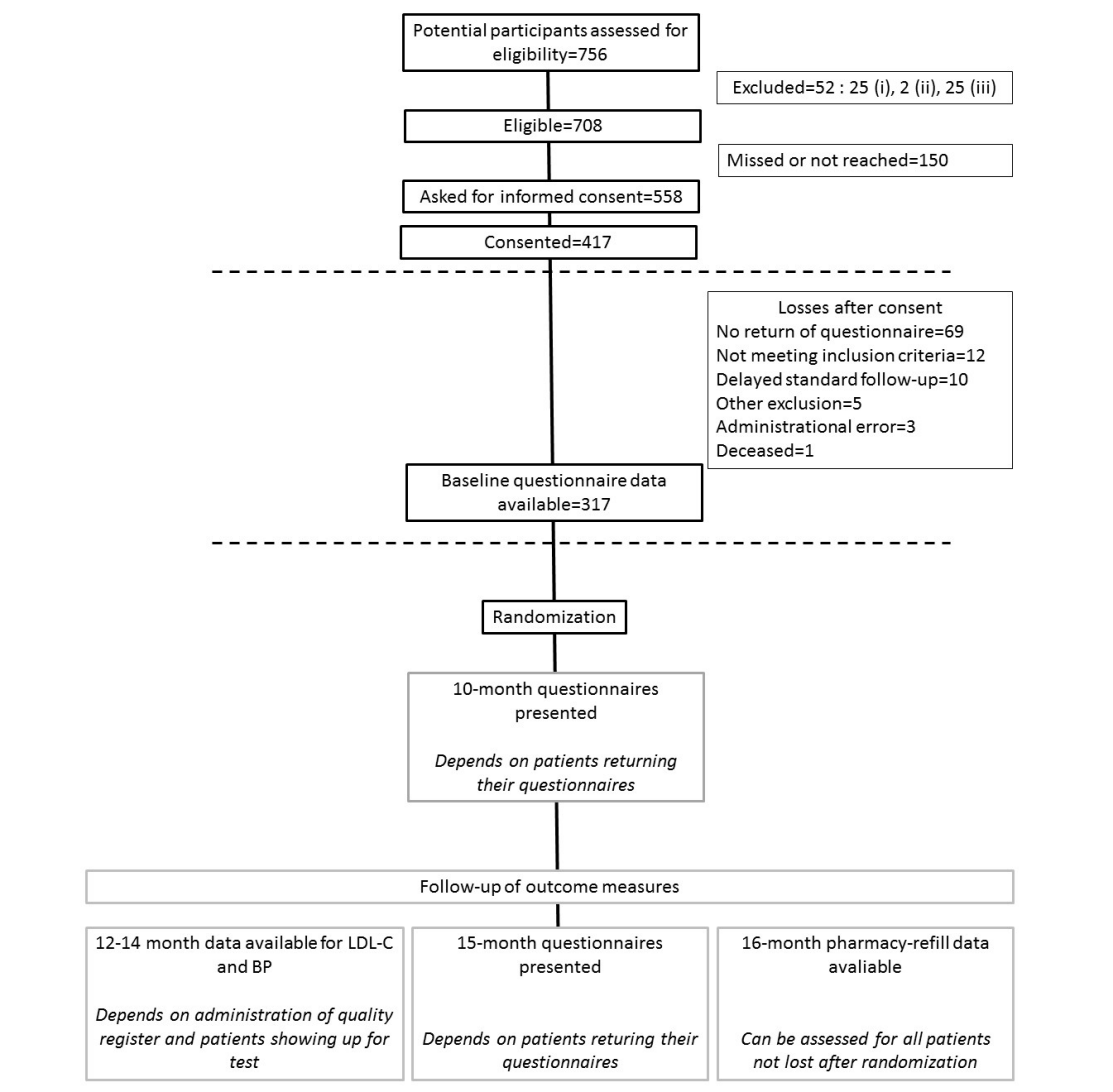
Flow diagram of study participants, status as of April 2017. For those excluded, (i) indicates cognitive impairment or any other condition making interviews or phone calls difficult; (ii) indicates nonparticipation in the standard follow-up at the out-patient clinic; and (iii) indicates prior participation in this study. LDL-C: low-density lipoprotein cholesterol; BP: blood pressure.

A total of 417 patients were included in the study before recruitment stopped in December 2016, see Figure 2. Unmet inclusion criteria were later identified for 12 patients, standard follow-up was delayed by more than 3 months for 10 patients, 5 patients were excluded for other reasons, 1 was deceased, and 3 were missed in administration, which resulted in 386 patients being sent baseline questionnaires. By April 2017, all had been approached and given baseline questionnaires and 317 patients had answered the questionnaires; 69 patients have withdrawn their participation by failing to return their baseline questionnaires.

Baseline characteristics have been registered in our database for the 234 subjects with baseline data collected before September 2016; these are described in Table 3. The mean (SD) age of the subjects is 68 (10.3) years and 73.9% (173/234) are male. About 70% (161/234) of the participants have had a myocardial infarction or other acute event, and about 30% (66/234) of participants have an earlier history of CHD. Medicines prescribed at discharge to these subjects can be seen in Table 4.

**Table 3:** Baseline characteristics of the first 234 subjects enrolled into the Motivational Interviewing and Medication Review in Coronary heart disease (MIMeRiC) study for whom complete baseline data are available.

Variable		All subjects
**Demographics**		
	Age (y), mean (SD)	68 (10.3)
	Male, n (%)	173 (73.9)
**Clinical history**		
	STEMI^a^, n (%)	68 (29.1)
	non-STEMI, n (%)	63 (26.9)
	Unstable angina, n (%)	30 (12.8)
	Chronic angina, n (%)	50 (21.4)
	Other reason for PCI^b^, n (%)	15 (6.4)
	History of CHD^c^, n (%)	66 (28.2)
**Beliefs about medicines**		
	Necessity score, mean (SD)	18.5 (3.8)
	Concern score, mean (SD)	12.9 (5.1)
	Accepting, n (%)	113 (48.3)
	Ambivalent, n (%)	75 (32.1)
	Neutral, n (%)	24 (10.3)
	Skeptical, n (%)	21 (8.9)

^a^STEMI: ST-elevation myocardial infarction.

^b^PCI: percutaneous coronary intervention.

^c^CHD: coronary heart disease.

**Table 4:** Medicines prescribed at discharge to the first 234 patients enrolled in the Motivational Interviewing and Medication Review in Coronary heart disease (MIMeRiC) study.

Medicine prescribed	At discharge, n (%)	New prescription^a^, n (%)
ASA^b^	202 (85.6)	126 (53.4)
Clopidogrel	81 (34.3)	69 (29.2)
Ticagrelor	119 (50.4)	118 (50.0)
Warfarin	20 (8.5)	8 (3.4)
ACEi^c^	119 (50.4)	80 (33.9)
ARB^d^	86 (36.4)	36 (12.7)
BB^e^	206 (87.3)	132 (55.9)
Statin	217 (91.9)	139 (58.9)

^a^Patients who have received a medicine for the first time.

^b^ASA: acetylsalicylic acid.

^c^ACEi: angiotensin converting enzyme inhibitor.

^d^ARB: angiotensin receptor II blocker.

^e^BB: beta-blocker.

## Discussion

This protocol describes the methodology for a study assessing the effectiveness of an intervention involving extended follow-up of the pharmacological treatment of patients with CHD using MI and medication review. Our randomized controlled trial acknowledges that optimal prescribing and monitoring of medications as well as high patient adherence is a prerequisite for adequate secondary prevention.

The aim of the intervention is to improve secondary prevention of CHD, and the effectiveness will be measured by assessing patient adherence as well as intermediate biological outcomes such as relevant treatment outcomes, perceived quality of life, and number of hospital admissions. We use two complementary adherence measures: self-report and pharmacy refill for the cholesterol drugs, which are directly linked to the primary outcome: LDL-C.

The design of this adherence intervention is based on a theoretical framework and it is the first trial of an intervention that uses beliefs about medicines to individualize the intervention protocol. Many adherence interventions have failed to assess or find long-term effects. Because this intervention follows the patient throughout the year after hospitalization for CHD and targets all patients regardless of adherence, we hope that it can prevent patients from discontinuing their medicines in the long term. It has been shown that the greatest loss in adherence (persistence) is during the first year and that patients who are persistent at 2 years continue to be adherent [47].

### Strengths and Limitations

Patients were recruited directly after their acute event or treatment for chronic CHD and were invited to participate regardless of age and comorbidities as long as they underwent the standard follow-up procedure at the clinic. However, because the intervention involves extra contacts with the hospital, some patients will decline participation; this could create a selection bias, especially among patients who live far from the hospital or patients with multi-morbidity or greater age. A limitation of this study is that it is conducted in one single hospital clinic. The findings may thus be generalizable to other clinics only in a limited manner.

The duration of follow-up is 12 months from the start of the intervention (ie, 15 months after discharge) for the outcomes measured by the study itself: adherence, quality of life, and hospital admissions. However, for practical reasons, we chose to use the follow-up at 12 months after discharge for measuring lipids and blood pressure, because these tests are already in place for the quality register for secondary prevention of myocardial infarctions. We acknowledge that 12 months’ follow-up might be too short to assess the effect on hospital admissions due to cardiovascular disease if the intervention primarily affects how patients manage their drugs in the longer perspective, and therefore, we aim to assess this outcome again after 3, 5, and 10 years.

The broad inclusion criteria and few exclusion criteria strengthen the generalizability of the study. The many outcomes of the study, from adherence and LDL-C to quality of life and hospital admissions, is another strength. Few adherence studies have used two adherence measures and are also designed to analyze a relevant clinical outcome, with follow-up of 1 year [11]. We also expect to have more than 80% of participants analyzed at follow-up [11].

### Protocol Amendments During Trial

After initiation of the study, we faced obstacles with our recruitment process, mainly due to patients not being identified for eligibility testing before discharge. We tried to remedy this by increasing the input from the research team, but after several months, without much difference in recruitment, we decided to change the procedure. This required the omission of a medication reconciliation that was initially part of the protocol for both study groups, as it was done at the inclusion phone contact before randomization. The reconciliation was appreciated by the nurses involved in the follow-up, but changing this meant little in the actual care of patients because the nurses were able to carry out a reconciliation during their consultation. After including the specialist nurses in the recruitment process, the enrollment rate increased markedly.

As described in the Methods section, the patent for atorvastatin expired during the study period, which resulted in more patients being treated with this drug as first-line treatment. This meant that more patients reached their treatment target without need of treatment assessment or changes in prescribing. We therefore recalculated our needed sample size based on more relevant presumptions. Another change affecting treatment was the release of the new American guidelines on treatment of blood cholesterol late in 2013 [48]. These guidelines no longer recommended a specific LDL-C treatment target for patients with CHD, but instead advocated high- or moderate-intensity statin treatment. This has affected how the cardiologists and pharmacists in Kalmar evaluate their patients’ treatment, even though national and European guidelines were not changed accordingly. We cannot assess how much this has affected the treatment of the study patients, but we assume that it lowers the motivation to reach an LDL-C target level.

During the study, we also learned that patients undergoing a coronary artery bypass graft operation were sometimes recruited into the study up to 6 months before their treatment actually took place. Because these are only a minority of the study participants, we will conduct a sensitivity analysis with this group excluded in the outcomes analysis.

### Clinical Implications

Patients today have concerns about their drugs because of what they read in the papers [49,50], what they hear from others, and the limited time for follow-up in their health care facility. They are left with no one to talk to about their concerns. Many patients suffer from adverse drug effects but do not contact their health care facility [51]. This results in a burden of health problems affecting their daily lives and causing worries, which possibly affect their quality of life. We propose that our model for extended follow-up will counteract this problem, but none of our outcome measures actually measure the impact of the intervention on an individual patient’s day-to-day living [52]. However, we believe that querying the patients on their beliefs about medicines possibly comes closest to providing information on how patients live with their medicines. We will therefore assess this and other measures of how the patients perceive the intervention in a process evaluation for which we publish a separate protocol [23].

## Abbreviations

BMQ-S: Beliefs about Medicines Questionnaire-Specific
CHD: coronary heart disease
CRF: clinical research form
EHR: electronic health record
LDL-C: low-density lipoprotein cholesterol
MI: Motivational Interviewing
MiMeRiC: Motivational Interviewing and Medication Review in Coronary heart disease
SEPHIA: secondary prevention after heart intensive care admission
